# Diphtheria

**Published:** 1884-06-20

**Authors:** W. M. Coleman

**Affiliations:** New Market, S. C.


### DIPHTHERIA.

Was called about four o'clock, a. m., April 6th, to see a girl nine years of age. The patient had an exudation covering the tonsils and pharynx; the tongue slightly yellow; temperature, 102; pulse 110. The lymphatic glands were greatly enlarged. The mother informed me that the disease had set in three days previous with a chill, followed by headache and fever. The voice of the patient had changed to a muffled sound. Had lost all appetite, and what food she ate was forced. My diagnosis was diphtheria. I used, as a spray, chlorate potash, gr. 15; water, ℥ j, to be repeated every two hours; chloride tincture of iron, ten-drop dose every hour. I then ordered a teaspoonful of whisky to be given every half hour. The patient made a rapid recovery.

The reason why I cite this case is to draw the attention of the profession to the use of whisky in this disease. I was very unsuccessful in the treatment of diphtheria until I adopted the whisky. Since then I have not lost a single case. The whisky may be increased to suit the condition of the patient.

*New Market, S. C.* W. M. COLEMAN, M. D.

